# Impacts of *Hibiscus esculentus* extract on glucose and lipid profile of diabetic rats

**Published:** 2015-05-19

**Authors:** Fatemeh Akbari, Najmeh Shahinfard, Mahmoud Mirhoseini, Hedayatollah Shirzad, Esfandiar Heidarian, Shabnam Hajian, Mahmoud Rafieian-Kopaei

**Affiliations:** ^1^Medical Plants Research Center, Shahrekord University of Medical Sciences, Sharekord, Iran; ^2^Clinical Biochemistry Research Center, Shahrekord University of Medical Sciences, Sharekord, Iran; ^3^Nickan Research Institute, Isfahan, Iran

**Keywords:** Diabetes mellitus, *Hibiscus esculentus*, Lipid profile, Hyperglycemia

## Abstract

**Introduction:** *Hibiscus esculentus* is capable to produce various molecules including phenolic and flavonoid compounds, phytosteroids with antioxidant property. Therefore, it has the potential to show antidiabetic activities.

**Objectives:** This study was aimed to evaluate the impacts of *Hibiscus esculentus* extract on glucose and lipid profile of diabetic rats. The flavonoid, flavonol and phenolic components, as well as antioxidant activity of *Hibiscus esculentus* was also evaluated.

**Materials and Methods:** In a preclinical study, 40 male Wistar rats were designated into four 10-member groups, i.e., control, diabetic control, diabetic *Hibiscus esculentus*, and diabetic glibenclamide. The Alloxan-induced diabetic rats received extracts orally for four weeks. Then, the serum biochemical factors were measured and compared by analysis of variance (ANOVA).

**Results:** Serum glucose, triglyceride (TG), cholesterol, and low density lipoprotein cholesterol (LDL-C) were significantly decreased and high density lipoprotein cholesterol (HDL-C) increased in diabetic *Hibiscus esculentus* rats compared to diabetic control ones (P < 0.05).

**Conclusion:** Improving the blood glucose and lipid profile in diabetic rats indicates that *Hibiscus esculentus* extract might be beneficial in diabetic patients.

**Implication for health policy/practice/research/medical education:**

Diabetes mellitus is one of the most widespread endocrine diseases. Considering that herbs are not without complications, due to the easy access, less side effects and toxicity, as well as proper cost are worthy of attention as suitable alternatives of synthetic drugs. Hibiscus esculentus plant is a rich source of bioactive molecules such as phenolic compounds, triterpenes and phytosterols with anti-oxidant activities. Therefor it is expected to be effective in improving glucose and lipid profile diabetes mellitus.

## Introduction

Diabetes mellitus is one of the most widespread endocrine diseases, referred to a group of metabolic disorders associated with high blood glucose (hyperglycemia) due to impaired insulin secretion or action (1).

It is usually associated with frequent urination, weight loss, and visual disturbance as the effects of overt hyperglycemia. The main reason related to diabetes death is cardiovascular diseases that are 2 to 4 times more common than in the general population. Also, the risk of cerebral infarction is increasing in these particular patients (1,2). Currently, the main and effective treatment for diabetes is the use of insulin and hypoglycemic agents. However, these compounds have numerous adverse effects such as increase of body fat deposit, atrophy of adipose tissue at the injection site and incidence of hypoglycemic shock (1-3). Furthermore, in long-term consumption they have not shown to be able to change the pathogenesis consequences of diabetic disabling complications. Hence, the need for finding effective combinations in diabetes treatment or prevention with fewer side effects is felt (4). Although herbs are not without complications (5), due to the easy access, less side effects and toxicity, as well as proper cost are worthy of attention as suitable alternatives of synthetic drugs. Recent researches have shown promising results in relation to the effect of these plants on the treatment of diseases (6,7).

## Objectives

The purpose of this study was to achieve a combination that can be effective on diabetes with minimal side effects. *Hibiscus esculentus* plant of the Hibiscus genus contains 220 species that have been scattered all over the world and is a rich source of bioactive molecules such as phenolic compounds, triterpenes and phytosterols with anti-oxidant activities. Also, *Hibiscus esculentus* plant is rich in carbohydrates, phytosterols, tannins and flavonoids. Therefore, it is expected to be effective in improving diabetes mellitus.

In this study, the effect of *Hibiscus esculentus* plant has been evaluated on glucose and lipid profile of diabetic rats.

## Materials and Methods

### Extraction

After providing and authentication the plant by the botanist, a herbarium specimen was prepared and deposited in the herbarium unit of Shahrekord University of Medical Sciences, Iran (Code No: 381).

In order to eliminate dust and trash, *Hibiscus esculentus* fruits were washed with water and placed in the shade to dry.

### Standardization of the extract

In order to standard the extract total flavonols, flavonoids and phenolic components as well as antioxidant activity of the extract were evaluated.

The phenolic compounds were measured equivalent to gallic acid consuming Folin-Ciocalteu colorimetry method (8). To do this, 0.1 ml gallic acid with different concentrations (12.5, 25, 50, 62.5, 100 and 125 ppm in methanol 60%) was transferred into a test tube containing 0.5 ml Folin-Ciocalteu (10%), as reactive agent. After 8 minutes, 0.4 ml sodium carbonate (7.5%) was added. The tubes were left at the laboratory temperature for 30 minutes and then were assayed in three intervals by a spectrophotometer (Unico UV 2010) at 765 nm wavelength. Then, 0.02 g of the extract was dissolved in 60% methanol, reaching 10 ml and the total phenolic components were measured from the read optical density in mg/g extract in gallic acid equivalent.

The total flavonoids were measured equivalent to rutin, by chloride aluminum colorimetry and rutin methods. To do this, one ml from different concentrations of rutin in methanol 60%. (25, 50, 100, 250 and 500 ppm) was transferred into test tubes and containing 1 ml of aluminum chloride 2% and 6 ml potassium acetate 5% was added. Then the optical density was read after 40 minutes at 415 nm wave length.

In order to measure the total levels of flavonoids in the extract, 0.02 g of the extracts was dissolved in methanol 60%, reaching 10 ml. Next, using chloride aluminum colorimetry the total level of flavonoids was measured. Though, instead of using the standard solution, 1 ml of the extract was added. The total flavonoid level was estimated in mg per one gram extract, equivalent to rutin (9).

### Measurement of flavonol compounds

Additionally the total flavonol was measured using chloride aluminum colorimetry and Rutin procedure, however the optical density level reading, was obtained after 2.5 hours at 440 nm wavelength (10).

### Procedure

Forty male Wistar rats weighting 200-250 g were accidentally divided into 4 groups of 10 and treated for 4 weeks as follows:

1. Control group: normal diet for 4 weeks
2. Diabetic control group
3. Diabetic group receiving turnip powder
4. Diabetic group receiving glibenclamide

### Induction of diabetes in animals

Rats were made diabetic by intraperitoneally (i.p.) injection of 120 mg/kg alloxan monohydrate (in normal) for three consecutive days. Criteria for being diabetic were the amount of blood glucose between 200-300 mg/dl (to prove diabetes, glucometer was used that is a useful technique for examination of initial stages of diabetes). Finally, the animals were made anesthetized and blood samples were collected for evaluation of biochemical parameters.

### Statistical analysis

In this study, analysis of variance (ANOVA) test and SPSS software version 11.5 were used for comparing means of investigating factors. All related charts were plotted in Excel software.

## Results

### The effect of Hibiscus esculentus powder on serum glucose

Three consecutive days administration of alloxan induced diabetes, so that the average of rats’ blood glucose increased from 7±97 to 308±490 mg/dl.

**Figure 1:**
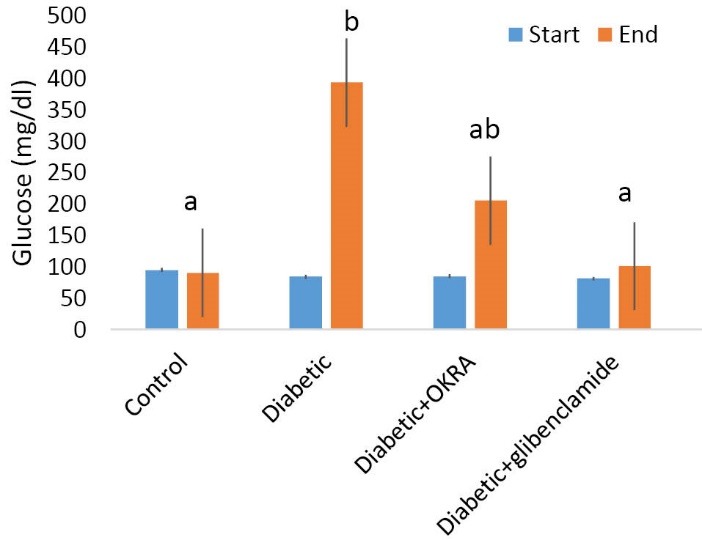
Comparison of blood glucose in investigated groups. The results are stated as mean ± SE (n = 10). ^a^ *P* < 0.05 in comparison to the related control group at the same time. ^b^ *P* < 0.05 at the end of period compared to beginning of period

The results of ANOVA showed that turnip powder extract could significantly reduce serum glucose in diabetic rats more than diabetic control group (*P*<0.05; Figure 1).

### Effect of Hibiscus esculentus powder on serum triglyceride

**Figure 2:**
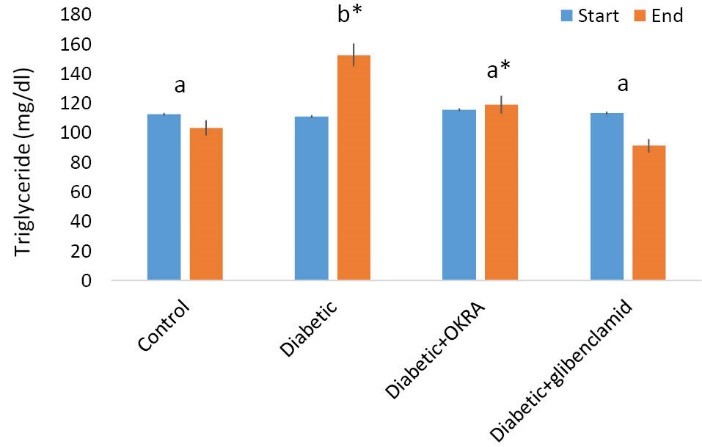
Comparison of serum TG in the investigated groups. The results are stated as mean±SE (n=10). ^a^*P*<0.05 in comparison to the related control group at the same time. ^b^*P*<0.05 at the end of period compared to beginning of period.

The serum triglyceride (TG) level in rats at the start (before treatment) and at the end of the experimental periods are shown in Figure 2. At the start of period, there was not any significant difference between the investigated groups. At the end of the period, in the treated group with Hibiscus esculentus, the amount of this factor was significantly reduced in comparison to diabetic control group (*P*<0.05).

### Effect of Hibiscus esculentus powder on serum cholesterol

**Figure 3:**
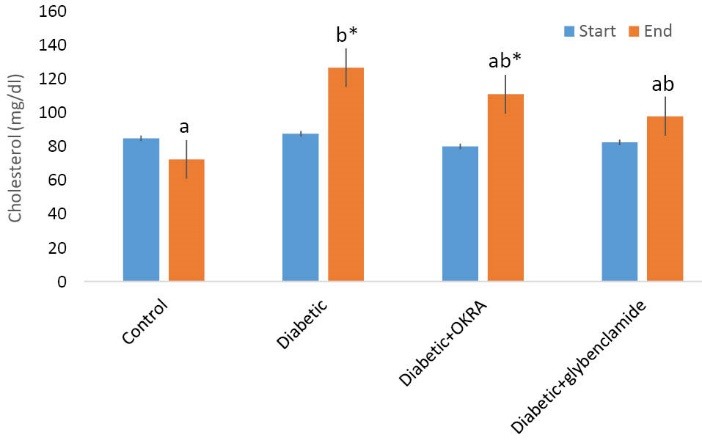
Comparison of serum cholesterol in investigated groups. The results are stated as Mean ± SE (n = 10). ^a^ *P* < 0.05 in comparison to the related diabetic control group at the same time. ^b^ *P* < 0.05 at the end of the period compared to the start of the experiment. * *P* < 0.05 in comparison to glibenclimide group at the same time.

The animal serum cholesterol level at the start (before treatment) and at the end of the experimental periods are shown in Figure 3. At the beginning of the period, no significant difference was observed between groups. At the end of the period in treated group with *Hibiscus esculentus*, the serum cholesterol level was significantly reduced in comparison to diabetic control group (*P*<0.05; Figure 3).

### Effect of Hibiscus esculentus powder on serum HDL-C

**Figure 4:**
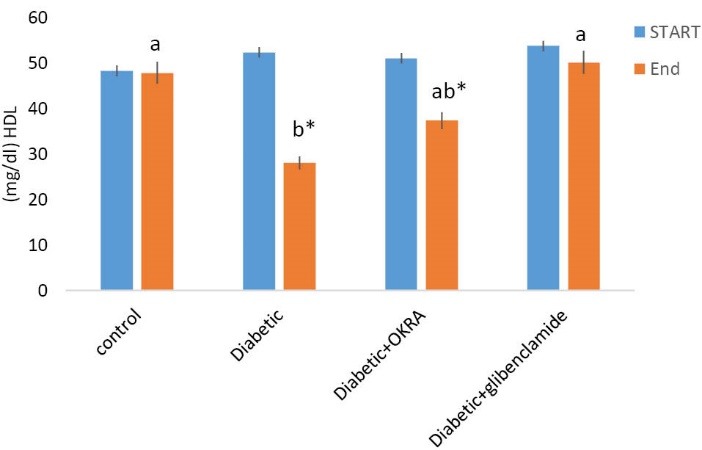
Comparison of HDL serum in investigated groups. The results are shown as Mean ± SE (n = 10). ^a^ *P* < 0.05 in comparison to the related diabetic control group at the same time. ^b^ *P* < 0.05 at the end of the period compared to the early period. * *P* < 0.05 in comparison to glibenclimide at the same time.

The serum high density lipoprotein cholesterol (HDL-C) level at the start and at the end of the experimental periods is shown in Figure 4. At the end of the period in the *H. esculentus* group, the amount of HDL-C was significantly increased compared to diabetic control group.

### Effect of Hibiscus esculentus powder on LDL-C serum level

**Figure 5:**
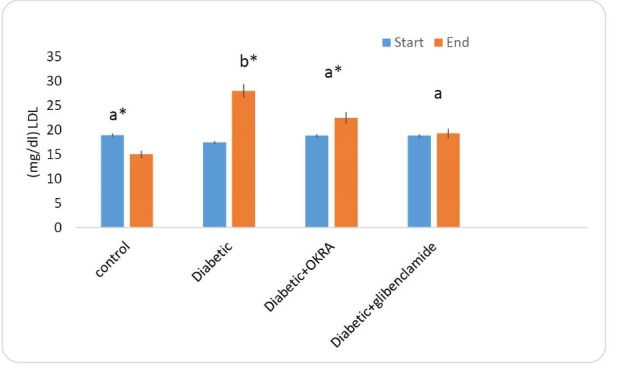
Comparison of serum LDL-C in the investigated groups. ^a^ *P* < 0.05 in comparison to the related diabetic control group at the same time. ^b^ *P* < 0.05 at the end of the period compared to the beginning of the period. * *P* < 0.05 in comparison to glibenclimide group at the same time.

The serum LDL-C level in the rats at the start and at the end of the experimental periods are shown in Figure 5. At the beginning of the period, no significant difference was observed between the investigated groups. At the end of the period, in the treated group with *Hibiscus esculentus*, the amount of this factor was significantly reduced compared to diabetic control group (*P*<0.05).

## Discussion

This study was aimed to evaluate the effects of *Hibiscus esculentus* extract on glucose and lipid profile of diabetic rats. By using alloxan monohydrate with a dose of 120 mg/kg in rats, we were able to make diabetes in rats. Alloxan causes the increase of plasma glucose with the destruction of the beta cells of the Langerhans islets. This finding is attuned with the findings of previous published ones (11,12).

Alloxan monohydrate mediates the toxicity of cells by increasing oxygen free radicals and induction of oxidative stress. Apoptotic cells are also killed by radicals. Due to structural similarities, alloxan is connected to the beta cells or inside these cells through sub-glucose receptors, destroying beta cells, selectively. Therefore, it will be used as a suitable tool in providing the experimental diabetes (13). Increase in blood glucose concentration is due to the destruction of pancreatic beta cells and impairment of insulin secretion.

Lack of insulin secretion causes the activation of phosphorylase enzyme. This enzyme causes the decomposition of glycogen to glucose phosphate. Hence, due to lack of insulin, the enzyme glucose phosphatase is activated and causes splitting of phosphate and glucose. This allows glucose to reenter the blood from the liver (14).

Increase in the concentration of blood glucose is associated with increase in the level of lipids, and plasma lipoproteins that in this relationship some body tissues, especially the liver due to absorption of blood free fatly acids, oxidation and its metabolic changeover to other materials, increased synthesis of cholesterol and phospholipids, and secretion of some types of lipoproteins into the blood are important. In addition, increased level of TGs and cholesterol in diabetic rats has been reported (15).

Increasing glucose level through alloxan will indirectly increase serum cholesterol, TG, LDL-C and decrease HDL-C levels (16). This may partly explain the unfavorable changes in serum lipids levels in diabetic rats in this research.

In the present study, the blood glucose was significantly reduced by glibenclamide. This drug is an antidiabetic medicine that can increase insulin release from pancreatic beta cells. In addition, the drug has insulin like effects on glucose metabolism, so, it may cause inhibition of glycogenolysis and gluconeogenesis. Thus, by inhibiting the above mentioned processes, glibenclamide reduce blood glucose level (17,18).

*Hibiscus esculentus* powder could significantly reduce the serum glucose concentration of diabetic rats compared to diabetic group. *Hibiscus esculentus* includes amine acids containing sulfur (19). These compounds have a direct function in reducing blood sugar and can enhance the effects of insulin on body and increase liver glycogen in rats and diabetic rabbits (20). This plant is also rich in carbohydrates, phytosterols, tannins, and flavonoids (21,22). Flavonoids have a variation of pharmacological effects such as protecting LDL-C from oxidation, anti-cancer, anti-diabetic, anti-allergic, liver protection, anti-inflammatory, and anti-tumor activities (23). Some flavonoids have inhibitory effect on the enzyme aldose reductase that has a role in the complications of diabetes (23,24). Although, some have hypoglycemic effect and increase glucose uptake in the muscles of normal health rats (25). Also, some flavonoids cause increase in insulin secretion and reduction in glucose in diabetic rats (26-28). *Hibiscus esculentus* plant also possesses beta-carotene (29) that can increase serum antioxidant level and thereby neutralizes free radicals, the main factors of pancreas destruction (27-30).

The results of this study indicate that in comparison to the diabetic control group, the treated group with *Hibiscus esculentus* powder caused significant reduction in cholesterol concentration, TG, and serum LDL-C and the significant increase in serum HDL-C. As it was mentioned, increase in blood cholesterol, TG, and LDL-C in diabetic rats might be due to increase in blood glucose. Reduction of these parameters in *Hibiscus esculentus* group can be due to direct effect on lipid profile or due to reduction of blood glucose (28).

This can be discussed in several directions. As mentioned, *Hibiscus esculentus* powder contains a large quantity of flavonoids. Flavonoids cause an increase in the number of LDL-C receptors on the side of liver cells through the impact on LDL-C receptor gene. LDL-C receptor binds to it by identifying apoprotein which absorb LDL-C into hepatocytes. Also, flavonoids increase the expression of LDL-C receptor in hepatic hepatocytes and have inhibitory effect on the synthesis of apolipoprotein B100 (apoB100) in liver cells and reduce LDL-C levels, hence, the polyphenols decrease the production of lipoproteins and increase their filtration in the hepatic cells (29,30).

On the other hand, the increase in insulin level, discussed in the previous section, may result in the activation of lipoprotein lipase in the capillary wall of adipose tissues. This enzyme breaks down TGs into fatty acids which can be absorbed and converted back into TGs again and be stored (27-30).

Diets rich in beta-carotene reduce blood cholesterol and prevent the development of fatty liver and inflammation in rats at the risk of atherosclerosis. Also, beta-carotene prevents accumulation of TGs in liver and plasma. But the exact mechanism of this effect is unknown because of the limited research on this subject (26-30).

## Conclusion

*Hibiscus esculentus* powder can reduce glucose level and improve lipid profile and might be beneficial in diabetic patients. However, further studies are recommended to elucidate its effects in diabetic patients.

## Acknowledgments

This paper was derived from MSc thesis of the first author.

## Authors’ contribution

All authors contributed equally to the paper.

## Conflicts of interest

The authors declared no competing interests.

## Ethical considerations

Ethical issues (including plagiarism, data fabrication, double publication) have been completely observed by the authors.

## Funding/Support

The authors would like to thank Medical Plants Research Center Shahrekord University of Medical Sciences for financial support (Grant# 1389-01-75-332).
